# Supplementary material for: Discomfort and Pain Related to Protective Mask-Wearing during COVID-19 Pandemic
Source: J Pers Med. 2022 Sep 1;12(9):1443. doi: 10.3390/jpm12091443 (PMC9501271; doi:10.3390/jpm12091443)

# How do you wear the mask?

Dear colleague, in order to cope with the SARS-CoV-2 virus emergency, last April 26th the DPDM entitled "Further implementing provisions of Decree-Law no. 6 of February 23rd, 2020, containing urgent measures for the containment and management of the epidemiological emergency from COVID-19, applicable throughout the national territory" came into force.
Based on this, the use of the mask has become of daily use especially for us healthcare workers.
We are asking for a few minutes of your time to respond to a survey we are conducting on the use of the mask and if this has caused you any problems.

1. Enter you age: ____________

2. Indicate your gender:

   - ☐ Female
   - ☐ Male

3. Indicate your job:
   - ☐ Physician
   - ☐ Rehabilitation Therapist (Physiotherapist, occupation therapist, speech therapist)
   - ☐ Nurse
   - ☐ Psychologist
   - ☐ Socio-Healthcare Worker

4. Approximately how many hours a day do you wear the mask? ____________

5. What kind of mask do you wear mostly?
   - ☐ Surgical
   - ☐ Cotton surgical
   - ☐ FFP2
   - ☐ FFP3

6. Do you wear two masks, one on top of the other? *(If you answered "no," move on to question n.8)*
   - ☐ Yes
   - ☐ No

7. If “yes”, which ones?
   - ☐ Surgical + FFP2
   - ☐ Surgical + FFP3
   - ☐ Surgical + cotton surgical
   - ☐ Surgical + Surgical

8. Have you had to change mask type due to the appearance of pain/irritation/sensitivity disorders?
   - ☐ Yes
   - ☐ No

9. How do you predominantly wear the mask?
   - ☐ Wearing the elastic bands behind the ears *(Skip to question n.13)*
   - ☐ using alternative "Ear Saving Strategies"

10. Why did you stop wearing the mask with the elastic bands behind your ears?
    - ☐ Skin lesions/irritation appeared in the upper part of the ear
    - ☐ Skin lesions/irritation appeared at the back of the ear
    - ☐ Skin lesions/irritation have appeared on the lower part of the ear, at the earlobe junction

11. What alternative "ear-saving" strategies do you use for wearing the mask?
    - ☐ Extensions or adjustable lanyards
    - ☐ Headband ties
    - ☐ Cotton ribbons
    - ☐ Other

12. How do you wear the "ear protector"?
    - ☐ Behind the neck, at the height of the nape of the neck (low position)
    - ☐ About halfway up the head (medium position)
    - ☐ At the top of the head (high position)

- I change position frequently so as not to "stress" the area on which the "ear protector" rests.

13. Using the mask, of any kind, did you notice if you had more headaches than usual?
    - Yes, I had more than usual
    - Yes, I started to get headaches
    - No, the number of headaches did not change *(Skip to question n.15)*
    - I do not have headaches *(Skip to question n.15)*

14. If you answered "yes" to the previous question, how would you define pain caused by a headache?
    - Pulsating pain
    - Circle-type pain in the head

15. Has using the mask brought you any other problems? *(You can select more than one answer)*
    - Neck pain
    - Temporo-mandibular occlusion
    - Teeth grinding at night
    - Skin irritations
    - Acne
    - Other: _______________________

16. Using the mask, did you experience a burning sensation, electric shock, pinprick and/or tingling?
    - Yes
    - No *(Skip to question n.19)*

17. If you answered "yes" to the previous question, where?
    - Forehead
    - Zygomatic region
    - Occipital region
    - Temporal region
    - Parotid region
    - Nasal region

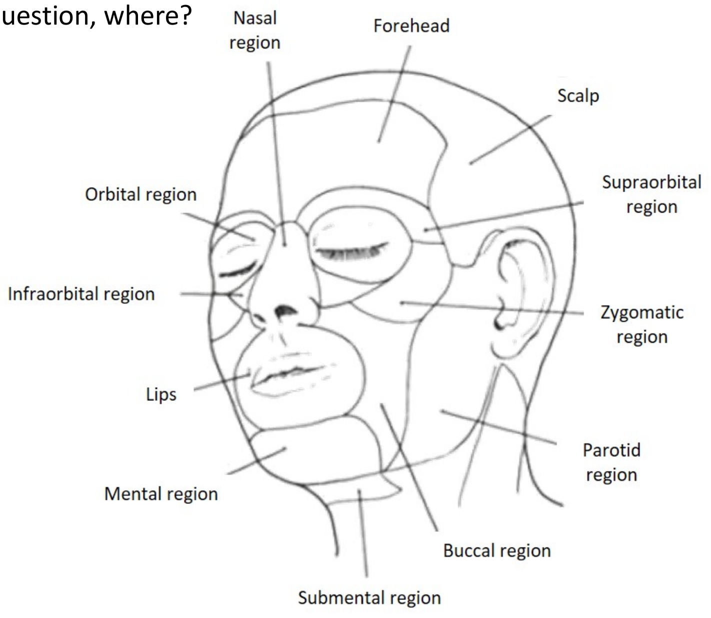

- ☐ Lips
- ☐ Submental region
- ☐ Other: ______________________

18. If you answered "yes" to question n.16, using the mask did you experience reduced sensation in your face, such as numbness, hypoesthesia, or anesthesia?
    - ☐ Yes
    - ☐ No

19. If you answered "yes" to the previous question, where?
    - ☐ Forehead
    - ☐ Zygomatic region
    - ☐ Occipital region
    - ☐ Temporal region
    - ☐ Parotid region
    - ☐ Nasal region
    - ☐ Lips
    - ☐ Submental region
    - ☐ Other: ______________________

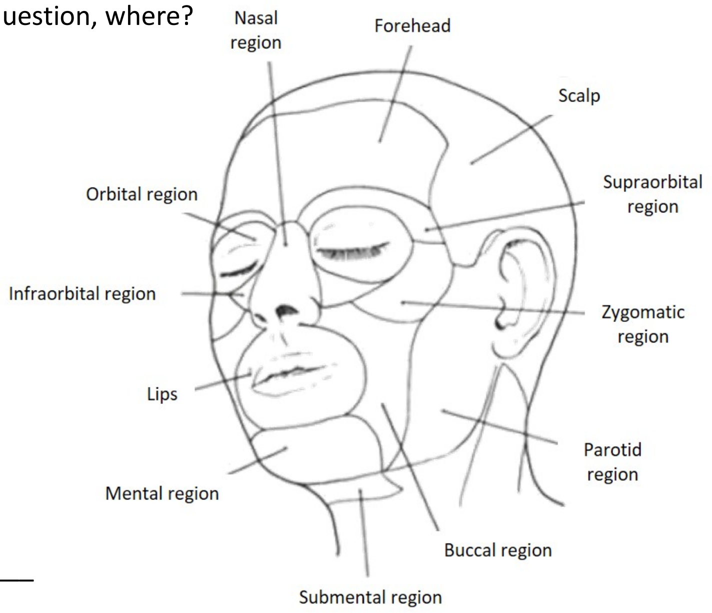

Supplement: Supplementary file 1 [file jpm-12-01443-s001.zip › jpm-1885357-supplementary.pdf]
